# Fabrication of Thin Film Composite Membranes on Nanozeolite Modified Support Layer for Tailored Nanofiltration Performance

**DOI:** 10.3390/membranes12100940

**Published:** 2022-09-27

**Authors:** Shabin Mohammed, Haya Nassrullah, Jamaliah Aburabie, Raed Hashaikeh

**Affiliations:** 1 NYUAD Water Research Center, Department of Engineering, New York University Abu Dhabi, Abu Dhabi P.O. Box 129188, United Arab Emirates; 2 Chemical and Biomolecular Engineering Division, Tandon School of Engineering, New York University, New York, NY 11201, USA

**Keywords:** TFC, polyamide, ball milling, nanozeolite, HY zeolite, nanofiltration, desalination

## Abstract

Thin-film composite (TFC) structure has been widely employed in polymeric membrane fabrication to achieve superior performance for desalination and water treatment. In particular, TFC membranes with a thin active polyamide (PA) selective layer are proven to offer improved permeability without compromising salt rejection. Several modifications to TFCs have been proposed over the years to enhance their performance by altering the selective, intermediate, or support layer. This study proposes the modification of the membrane support using nanozeolites prepared by a unique ball milling technique for tailoring the nanofiltration performance. TFC membranes were fabricated by the interfacial polymerization of Piperazine (PIP) and 1,3,5-Benzenetricarbonyl trichloride (TMC) on Polysulfone (PSf) supports modified with nanozeolites. The nanozeolite concentration in the casting solution varied from 0 to 0.2%. Supports prepared with different nanozeolite concentrations resulted in varied hydrophilicity, porosity, and permeability. Results showed that optimum membrane performance was obtained for supports modified with 0.1% nanozeolites where pure water permeance of 17.1 ± 2.1 Lm^−2^ h^−1^ bar^−1^ was observed with a salt rejection of 11.47%, 33.84%, 94%, and 95.1% for NaCl, MgCl_2_, MgSO_4,_ and Na_2_SO_4_ respectively.

## 1. Introduction

Rapid population growth and industrialization in recent years have resulted in freshwater scarcity, which is expected to be escalated further in the upcoming years [1,2,3]. Moreover, technological advancement and ever-increasing stringent water quality standards put forward by authorities also necessitate the need for the development of novel treatment technologies to overcome these challenges [4,5]. Recent years witnessed a breakthrough in water purification and reuse with the introduction of membrane-based technologies [6,7]. Particularly, nanofiltration (NF) has gained a predominant space among various membrane-based technologies for desalination and wastewater treatment due to its relatively lower energy requirement [8]. Over the years, several membrane materials, including polymeric, ceramic, and other inorganic materials, have been explored for nanofiltration applications [9]. However, polymeric materials are still considered to be a favorite candidate due to their relatively cheaper production cost, flexibility, and facile synthesis routes [10]. Highly dense polymer structures can provide sub-nanometer selectivity, making them an excellent candidate for molecular level separation, particularly for NF membranes.

The most conventional design of polymer-based NF membranes comprises a thin film composite (TFC) structure fabricated by the interfacial polymerization (IP) reaction where a thin selective layer is fabricated on highly porous support [11]. Such a composite structure facilitates the optimization of each layer independently to obtain the desired performance. However, it is well established that the permeability and selectivity of such TFC membranes are mostly governed by the active polyamide (PA) layer, and most of the research has been focused on modifying the properties of the top selective layer. For instance, inspired by the promising results observed by Jong et al. by incorporating zeolites in the organic phase [12], several researchers proposed the modification of the selective layer using various nanomaterials [13,14,15,16,17,18]. Nevertheless, the addition of nanofillers in the selective layer often results in decreased selectivity due to the hindrance in cross-linking and weak adhesion of nanoparticles with the polymer matrix [19,20]. Alternatively, researchers have explored the possibility of introducing an intermediate layer between the support and PA selective layer to enhance the performance of TFCs [21,22,23,24,25]. Nevertheless, this might lead to the detachment of the selective layer over longer operation due to the poor adhesion of the PA layer with the selective layer; as in most cases, the intermediate layer is not connected by a chemical bond [26]. Therefore, it is more efficient to enhance the performance of TFCs without sacrificing selectivity and structural stability through an alternative strategy.

Recent studies have revealed promising results in modifying the support layer to enhance the overall performance of TFCs wherein suitable materials are incorporated into the support [27,28,29,30,31]. As modified supports exhibited improved hydrophilicity [27,28,29,30,32], porosity [27,28,29,30,31], surface charge [32], mechanical strength [31,32], and permeance [27,29], depending upon the nanoparticle characteristics, thereby significantly influencing the overall membrane performance. For example, MoS_2_ nanosheets have been successfully incorporated into the support layer to improve its hydrophilicity and porosity, which subsequently enhanced the overall performance of the TFC membrane [27]. Accordingly, the permeance of the TFC membrane was doubled without losing the membrane selectivity by incorporating just 0.75% MoS_2_ in the support. Similarly, incorporating carbon nanotubes in the support layer was also reported to enhance the overall performance due to improved porosity and permeability of the support, consequently leading to lower support resistance [28,30]. In a different approach, supports blended with hydrophilic polymers were also shown to exhibit better hydrophilicity and improved porosity, thereby achieving a smaller selective layer with superior performance [33,34]. These studies indicate that utilizing novel materials or chemicals that can modify the support properties without altering the selective layer can be effectively used for developing TFCs with superior performance.

Zeolite-Y with faujasite (FAU) structure has been demonstrated to be a potential candidate for membrane fabrication and modification owing to its hydrophilicity and unique inter-crystalline pores [35,36,37,38,39,40]. Additionally, membrane additives with reduced dimensions are favorable candidates for membrane modification due to favorable properties such as improved hydrophilicity [41,42], better distribution [43], and higher surface charge [39]. In line with this, recent studies in our group have shown that membrane modification using nanosized Y-type zeolites can enhance the performance of desalination and ultrafiltration membranes by contributing positively toward hydrophilicity and microstructure [39,40]. Therefore, in this study, we present the use of nanozeolites prepared by the wet ball milling process for the modification of Polysulfone (PSf) support, which is subsequently used in PA membrane fabrication. We embedded nanozeolites in different concentrations (0%, 0.05%, 0.1%, 0.15%, and 0.2%) into the support layer, thereby achieving varying hydrophilicity, porosity, and water permeance. Our results showed that the NF performance of the PA membranes could be tailored by incorporating nanozeolites of different concentrations in the support layer.

## 2. Materials and Methods

### 2.1. Materials

PSf pellets (average Mw ~35,000 g mol^−1^), Polyvinylpyrrolidone (PVP) powder (Mw ~55,000 g mol^−1^), n-Methyl-2-Pyrrolidone (NMP), Piperazine (PIP) (99%), 1,3,5-Benzenetricarbonyl chloride (TMC), Sodium Chloride (NaCl), Magnesium Chloride (MgCl_2_), Magnesium Sulfate (MgSO_4_), Sodium Sulfate (Na_2_SO_4_), and Ethanol were supplied by Sigma-Aldrich. Zeolite-Y (CBV 720) was purchased from Zeolyst International, while carbon nanostructures (CNS) were obtained from Applied Nanostructured Solutions LLC. All chemicals were used as received without any further purifications and PIP aqueous solutions were prepared using Milli-Q water.

### 2.2. Nanozeolite Preparation

Nanozeolites were prepared by a unique ball milling process developed in our group, which is reported in [44]. Briefly, micro-sized commercial zeolite-Y particles were initially mixed with CNS (mass ratio of 3:1) in a solution containing equal volumes of ethanol and deionized water (DI). The obtained thick paste-like dispersion underwent ball milling in zirconia jars with zirconia balls for a duration of 1 h at 1000 rpm. Ball milling was carried out using an E-max high-energy ball mill machine from Retsch, Germany. The product obtained was centrifuged at 4000 rpm for 10 min to isolate the desired nanozeolites (bottom part). Subsequently, the nanozeolites/CNS composite collected from the bottom part was dried at 80 °C overnight, followed by calcination at 610 °C for 5 h to remove CNS and recover nanozeolites. The prepared nanozeolites were stored in powder form at ambient conditions for further characterization and membrane fabrication.

### 2.3. Fabrication of Membrane Supports

Membrane supports were fabricated by the commonly employed wet phase inversion method. Initially, polymer casting solution was prepared by dissolving 3% of PVP and 17% of PSf in NMP at 60 °C accompanied by overnight stirring. The concentration of PVP was fixed at 3% based on some preliminary investigations to achieve support with optimum flux and pore characteristics. The polymer solution was cast on a glass plate using a semi-automatic casting machine (PMI Porous Materials Inc. Model BT FS- TC, US) at a constant shear rate of 200 s^−1^ and a casting thickness of 200 µm. Subsequently, the glass plate was immersed in a DI water bath at room temperature to induce phase inversion. The resulting membrane supports were rinsed and soaked in DI water for 3 days. The water bath was replaced every 24 h to ensure the complete removal of excess solvents and to minimize the effect of residual PVP. The procedure remained the same in the case of substrates containing nanozeolites, except that nanozeolites with varying concentrations were initially dispersed in NMP with the aid of a probe sonicator (Q Sonica Ultrasonic Processor) before the addition of PVP and PSf. S-0%, S-0.05%, S-0.1%, S-0.15%, and S-0.2% represent the different support layers prepared with varying nanozeolite concentrations, whose parameters are listed in Table 1.

### 2.4. Fabrication of PA Layer

**Figure 1:**
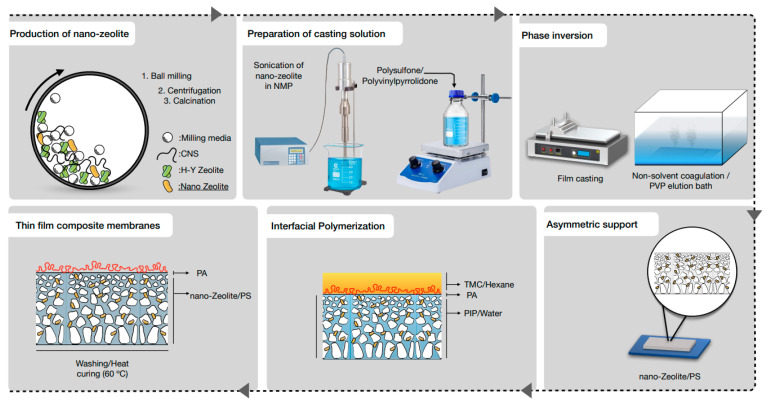
Membrane fabrication steps.

The PA layer was fabricated by IP reaction of aqueous and organic phases containing monomers PIP and TMC, respectively. The concentration of PIP and TMC were fixed as 2% (in Milli-Q water) and 0.25% (in hexane) based on some recent reports [34,45]. Firstly, supports were mounted onto a custom-made plate and frame (presented in Figure S1 in Supplementary Materials) with the top layer exposed to air. Thereafter, the top layer is treated with 25 mL of PIP solution for 1 min followed by the removal of excess solution with the help of an air gun and filter paper. Subsequently, the supports coated with PIP were contacted with 15 mL TMC solution for a short duration of 10 s to carry out the polymerization reaction. Subsequently, the membrane was cleaned with hexane to remove unreacted monomers and heated at 60 °C for 3 min before storing in DI water. Membranes corresponding to supports S-0, S-0.05, S-0.1, S-0.15, and S-0.2 were labeled as M-0, M-0.05, M-0.1, M-0.15, and M-0.2. The entire membrane fabrication steps starting from nanozeolite preparation, are illustrated in Figure 1.

### 2.5. Characterization

Morphology and particle size of the nanozeolites were analyzed using a scanning electron microscope (SEM) (FEI Quanta 450 FEG) at 10 kV. Samples were prepared by depositing dilute aqueous dispersion of nanozeolites on a silicon sample holder, followed by a metal coating. Any variations in the crystal structure due to ball milling were examined by X-ray diffraction (XRD) patterns recorded in the 2θ range of 2−80° at room temperature using X-ray Powder Diffraction (Malvern™, Empyrean 2, Malvern, UK) at a scan rate of 2° min^−1^. Fourier transform infrared (FTIR) spectra were recorded in a range of 500 cm^−1^ to 4000 cm^−1^ using a spectrometer (Thermo scientific™, Nicolet iS5 with iD7 ATR accessory) to investigate any changes in the chemical structure of the nanozeolites. The pore size distribution of nanozeolites was analyzed using the N_2_ adsorption experiment (NOVA^®^ 4200e Quantachrome Instruments). Samples were degassed at 200 °C for 12 h before the measurement to ensure the removal of moisture and any gases adsorbed.

All supports and membranes were air-dried before characterization. The porosity of all membrane supports was estimated by a gravimetric method where an average of three measurements has been reported for reliable results. All samples were exposed to the wetting liquid (Silwick™; surface tension = 22.1 dynes cm^−1^) for a period of 24 h after which the dry and wet weights were used to calculate the porosity based on the method described in [46]. Changes in hydrophilicity of the supports due to the varying nanozeolite concentration have been investigated by analyzing the static water contact angle using the sessile drop method (DSA100, Krüss GmbH, Hamburg, Germany). Measurement was carried out by placing water droplets of 2 µL in volume using a 0.51 mm diameter needle syringe. Values obtained by measurement at five different points were used to calculate an average contact angle for each sample. Mechanical properties of the membrane supports were investigated using a universal testing system (5965 model, Instron, US), where samples are subjected to 50 N load. For this, samples were cut into a standard dumbbell shape with overall dimensions 65 mm × 10 mm (length × width) and a gauge of 16 mm × 3.5 mm (length × width) using Ray/Ran Hand Operated Test Sample Cutting Press (RDM test equipment, UK).

The surface and cross-sectional morphology of all the supports and membranes were analyzed using SEM (FEI Quanta 450 FEG), where imaging was carried out at a higher voltage of 15 kV. Samples after metal coating were mounted on sample holders using carbon tape. The distribution of nanozeolites on the support surface has been confirmed by energy dispersive spectroscopy (EDS) integrated with the same SEM instrument. Measurements were carried out with the help of an EDS detector with a take-off angle 36° and at a working distance of 10 mm. Samples were exposed to electron beams at 10 kV with a spot size of 3. The surface chemistry of all samples was evaluated by capturing FTIR spectrum in a similar fashion explained previously. Surface charges of all the PA membranes were investigated by measuring the surface zeta potential at neutral pH using ZetaSizer (ZEN3600, Malvern Panalytical, UK) with polystyrene tracer solution.

### 2.6. Performance Testing

Pure water permeance of membrane supports and NF performance of all TFC membranes were evaluated at a pressure difference of 2 bar and 6 bar, respectively, using a bench-top stainless steel dead-end filtration set-up supplied by Sterlitech USA. The schematic sketch of the filtration unit is shown in Figure S2. All membranes were cut into circles of 2.5 cm diameter for testing, bringing down the effective membrane testing area to 3.14 cm^2^. To minimize the effect of initial compaction, membranes underwent compaction for 30 min at the testing pressure. The volume of the testing solution was kept constant at 150 mL and accompanied by magnetic stirring to minimize concentration polarization effects. The molecular selectivities of membranes towards monovalent and divalent salts were determined by measuring the rejection of aqueous salt solutions of NaCl, MgCl_2_, MgSO_4,_ and Na_2_SO_4_ with a concentration of 1000 ppm. Permeance and rejection were determined using the formulas given below, where salt concentration was quantified using a conductivity meter. An average of three reproducible values were used to ensure the credibility of the results.

(1) $$Permeance=\frac{V}{A.∆t.∆P}$$

where *V* is the volume of permeate, ∆t is the time needed to collect, *A* is the membrane testing area, and ∆P is the transmembrane pressure during the testing.

(2) $$Rejection=1-\frac{C_{P}}{C_{f}}$$

where *C_p_* and *C_f_* are the conductivity of permeate and feed, respectively.

## 3. Results and Discussions

### 3.1. Nanozeolite Preparation

**Figure 2:**
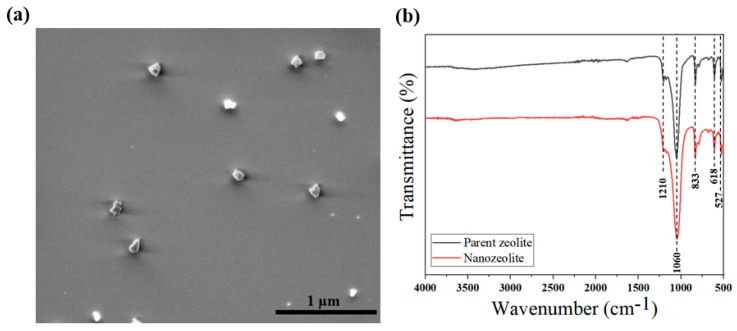
(**a**) SEM image of nanozeolites (**b**) comparison of FTIR spectrum of nanozeolite with parent zeolite.

Figure 2a shows the typical morphology of nanozeolites obtained after the ball milling process. More than half of the particles were in a size range of 75 nm to 125 nm with a size distribution as presented in the supplementary Figure S3. Nanozeolites exhibited irregular morphology resulting from the grinding, which is consistent with the previous studies that followed a similar procedure [39,44]. Figure 2b shows the comparison of the FTIR spectrum obtained for nanozeolites to that of the parent zeolites where similar spectra were observed. FTIR spectrum of nanozeolites revealed characteristic peaks of Y-type zeolite structure at 527, 618, and 833 cm^−1^ along with the common zeolite peaks (1060 and 1210 cm^−1^) [47,48]. This not only assures that zeolites are chemically stable at the calcining temperature and ball milling conditions but also confirms that no components from the CNS remained after the calcination step. Further, it is critical to ensure that the porous structure in the zeolites is preserved even after the harsh grinding conditions and elevated thermal treatment steps. For this, a nitrogen adsorption study was carried out where pore size distribution was estimated using the DFT method, which is shown in the inset of Figure S4. It is evident that nanozeolites preserved their porous structure and exhibited pore distribution and adsorption isotherm matching with the previous reports [36,49,50]. Finally, to identify any crystal changes during nanozeolite formation, XRD patterns were also compared with the parent zeolite, as shown in Figure S4. Both samples exhibited identical peaks characterized by Y-type zeolites at $6.28^{{}^{\circ}}$, $12.05^{{}^{\circ}}$, $15.86^{{}^{\circ}}$, $20.65^{{}^{\circ}},$ and $23.96^{{}^{\circ}}$ representing crystal planes (111), (311), (331),(440), and (533), respectively. All the above results confirm that the microstructural properties of the parent zeolites are preserved during the nanozeolite preparation.

### 3.2. Characterization and Performance of Membrane Support

**Figure 3:**
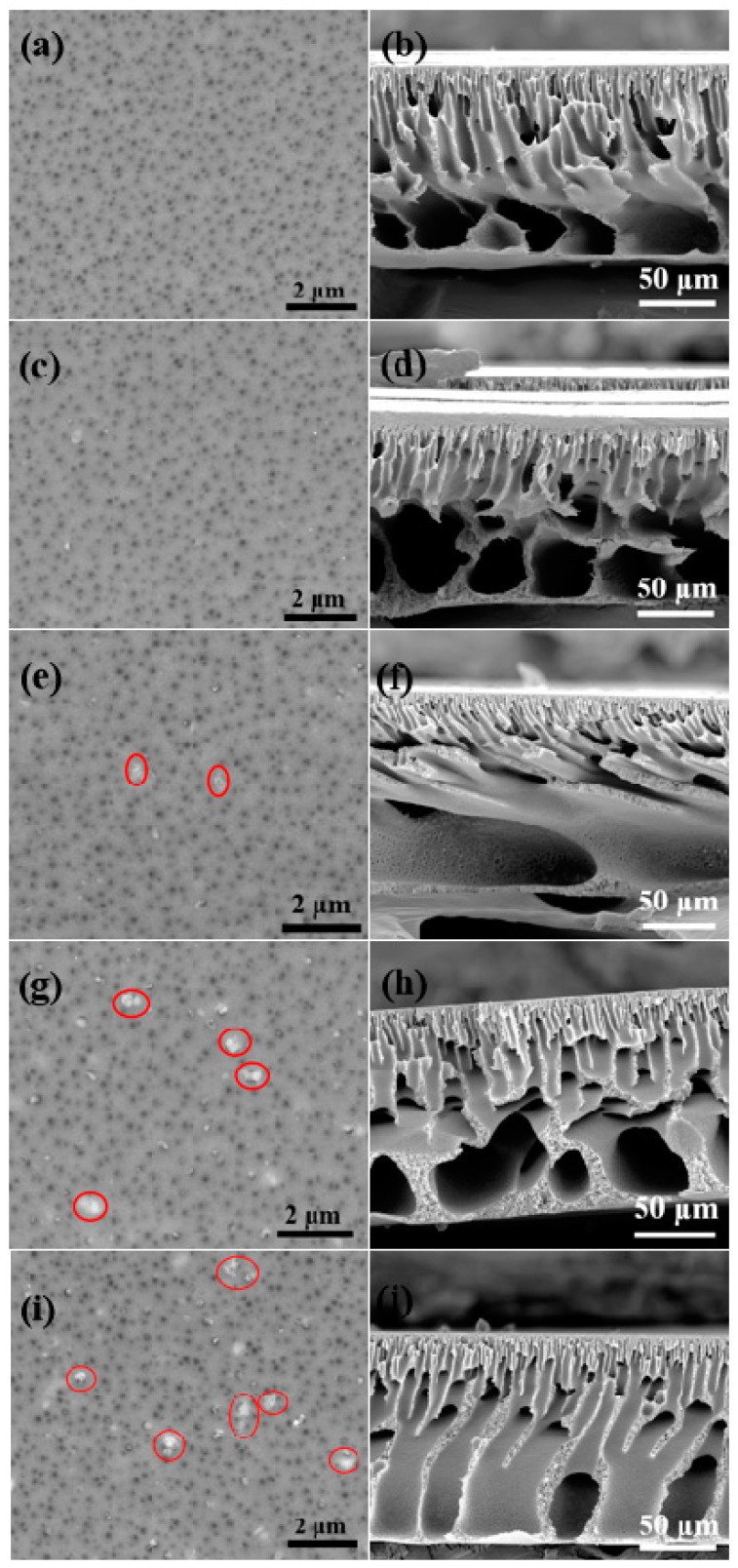
Surface and cross-sectional images of membrane supports: (**a**,**b**) S-0, (**c**,**d**) S-0.05, (**e**,**f**) S-0.1, (**g**,**h**) S-0.15, and (**i**,**j**) S-0.2.

The surface and cross-section morphology of all the membrane supports with varying nanozeolite content was analyzed through SEM, which is presented in Figure 3. Large macro-voids were present on the surface of all supports irrespective of the nanozeolite concentration. For instance, even the control sample without any nanozeolites exhibited large pores ranging from 50 nm to 200 nm, which is possibly due to the presence of PVP in the casting solution. We used PVP with a smaller molecular weight, thus enabling faster leaching into the water bath, whereas denser PVP often limits the diffusion rate leading to a denser top surface [51]. Surface images clearly showed the gradual increase in the presence of nanozeolite on the surface of the supports by the addition of nanozeolite in the casting solution. In general, the size of nanofillers was apparently larger relative to the size distribution reported from the SEM of the nanozeolite sample. A similar observation has been presented in previous studies where nanoparticles are reported to be aggregated when dispersed in a polymer matrix [52]. Particularly, zeolites with high surface energy are prone to form clusters of larger size to minimize their surface area [53]. Moreover, an even larger aggregation of nanozeolites could also be seen at higher loading, as indicated by red circles, which might adversely affect the permeance. The distribution of nanozeolites on the support surface has been further confirmed through the EDS spectrum and elemental mapping shown in Figures S5 and S6. As expected, no Si peaks were not detected in the EDS spectrum of the control sample. Additionally, M-0.05 also did not exhibit any Si peak, although its presence has been clearly evident in the SEM images and elemental mapping. This could be due to the well-dispersed nanozeolites in the polymer matrix at lower concentrations [39].

**Figure 4:**
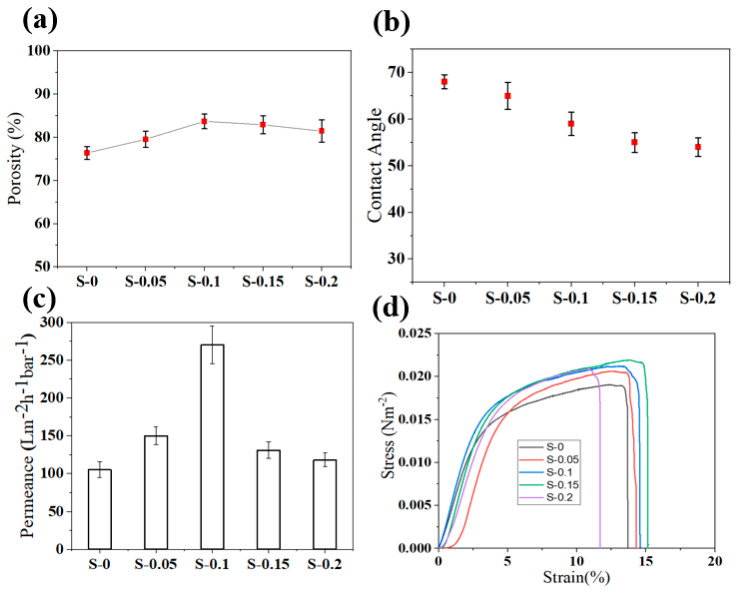
(**a**) Porosity, (**b**) water contact angle, (**c**) permeance, and (**d**) stress–strain curve for all the membrane supports.

Further, it is evident that the addition of nanozeolite in the casting solution had a significant impact on the overall porosity and surface pores distribution of the supports. The surface pore size gradually increased with the addition of nanozeolites (Figure S7). Similarly, the porosity of the supports also increased significantly until a concentration of 0.1% and thereafter registered a slight decrease, as presented in Figure 4a. This variation in the porosity and surface pores size distribution can be attributed to the increase in viscosity and hydrophilicity of the casting solution by the addition of nanozeolites. The presence of hydrophilic nanozeolites in the casting solution enables instantaneous de-mixing during phase inversion [54]. Consequently, diffusion of water into the polymer matrix is enhanced, thereby improving the porosity and pore size of the supports. On the other hand, the addition of nanozeolite progressively increases the viscosity of the casting solution, which results in a delayed de-mixing and thereby lowers the solvent exchange contributing to decreased porosity [55]. The effect of viscosity seems to be less significant in the case of pore size distribution, as pore size seems to be progressively increasing with the addition of nanozeolites, possibly due to a higher impact of the hydrophilic nanozeolites clusters distributed at the surface at higher loading. Meanwhile, cross-sectional images revealed that all the membrane supports, irrespective of the nanozeolite presence, exhibited asymmetric structure with a relatively denser top layer and a porous sub-layer. Large macro-voids on the surface and as well as on the cross-section are highly desirable for lower transport resistance, making them favorable to be used as a substrate for PA membranes.

Surface hydrophilicity is an important parameter that not only influences the permeability of the support but also the mechanism of PA formation. Hydrophilic supports are favored for defect-free PA formation. Therefore, in order to assess the variations in surface wettability of the support layer due to the addition of nanozeolites, the static water contact angle was measured, which is presented in Figure 4b. An increase in nanozeolite content was observed to be accompanied by a drop in contact angle due to the hydrophilic nature of the nanozeolites. As expected, the contact angle recorded by neat PSf support S-0 was 68° which decreased gradually to 54° for S-0.2. During the phase inversion process, hydrophilic particles migrate to the interface of the polymer and are non-solvent to minimize the interphase energy, which is also evidenced in the SEM images [56]. Particularly, FAU zeolites with a high Si/Al ratio used in this study are characterized by a relatively higher affinity toward water [40]. In addition, it is worth mentioning that a higher rate of diffusion is expected in the case of nanofillers due to their smaller size [54,56]. Accordingly, these nanozeolites at the surface contribute to a lower contact angle.

Figure S8 shows the FTIR spectra of all the supports. All spectra appeared to be identical irrespective of the nanozeolite concentration. Samples exhibited peaks corresponding to the chemical functional groups in the PSf, including 1149 cm^−1^ corresponding to O-H deformation, 1581 cm^−1^ referring to aromatic rings; 1488, 1402, 1242, and 1102 cm^−1^ from the stretching vibration of C–S, C–H, C–O–C, and C–O, respectively [57]. Peaks lower than 1085 cm^−1^ could be attributed to the benzene rings [58]. Additionally, a weak peak could also be seen at 1665 cm^−1^ in all the spectra that can be assigned to the amide group existing in the PVP molecules entrapped in the polymer [59]. None of the peaks corresponding to nanozeolites revealed in Figure 2b were observed even for S-0.2, possibly due to the low concentration of nanozeolites with respect to PSf that led to the masking of zeolite peaks; however, these results suggest that nanozeolites embedded in the polymer matrix did not alter the chemical structure of the polymer chains.

The permeability of the support is one of the most important parameters that determine the overall performance of the TFC membrane. A highly permeable support layer is favorable for lower transport resistance; therefore, we evaluated the pure water flux of all the membrane supports, which is presented in Figure 4c. Permeance was seen to be increasing with the addition of nanozeolites until S-0.1 and thereby drastically decreased with the nanozeolite concentration. While neat PSf supports offered pure water permeance of 105 ± 10.2 Lm^−2^ h^−1^ bar^−1^, M-0.1 exhibited a remarkably higher permeance of 270 ± 25.2 Lm^−2^ h^−1^ bar^−1^. This increase in flux could be mainly ascribed to the improved hydrophilicity, surface pore size, and porosity. However, M-0.15 and M-0.2 offered significantly lower flux compared to M-0.1 despite having slightly better pore size and comparable porosity. This can be explained by the severe aggregation of nanozeolites at higher loading, as evidenced by SEM images that lead to pore blocking [60,61]. Despite the favorable performance offered by the nanozeolite embedded support layer, it is important to assess the influence of nanofillers on their mechanical stability. Figure 3d shows the stress–strain curve obtained from the simple test for various supports. It is evident from the results that the mechanical stability is observed to be slightly improved with the addition of nanozeolites.

### 3.3. Characterization and Performance of PA Membranes

**Figure 5:**
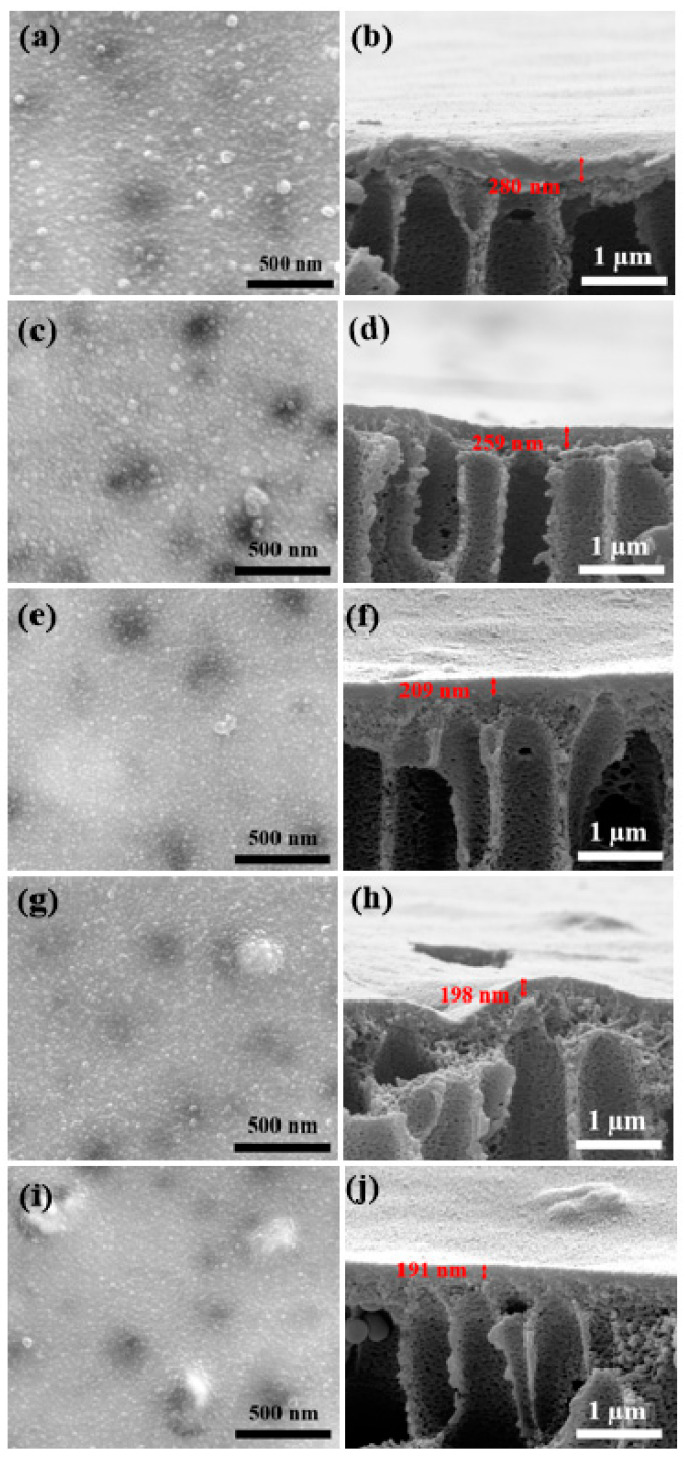
Surface and cross-sectional images of PA membranes on various supports (**a**,**b**) M-0, (**c**,**d**) M-0.05, (**e**,**f**) M-0.1, (**g**,**h**) M-0.15, and (**i**,**j**) M-0.2.

**Figure 6:**
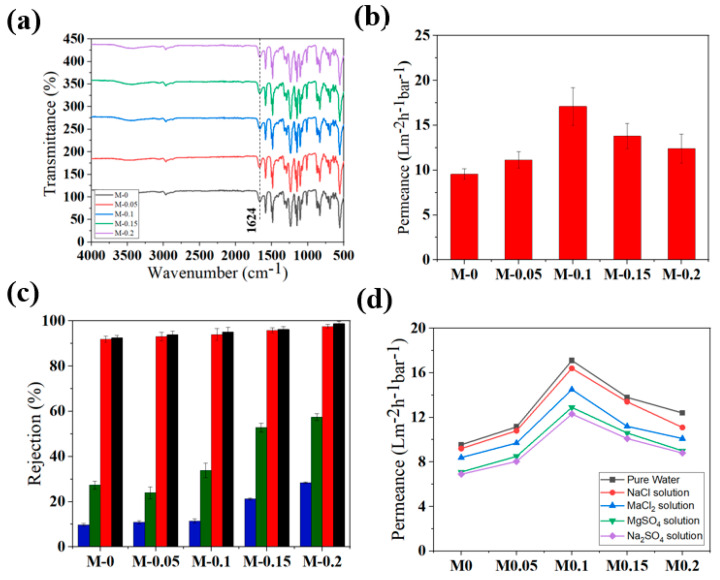
A comparison of (**a**) FTIR spectrum, (**b**) pure water permeance, (**c**) separation performance, and (**d**) salt solution permeance of NF membranes.

PA layer was formed on all the membrane supports S-0, S-0.05, S-0.1, S-0,15, and S-0.2 by the IP reaction between PIP and TMC and were labeled, respectively, as M-0, M-0.05, M-0.1, M-0.15, and M-0.2. Figure 5 presents the SEM images of the surface and cross-section of all the nanocomposite membranes. Surface images of all the membrane supports (presented in Figure 3) exhibited a highly porous morphology, whereas after PA formation, a denser top layer was observed. The membrane surface was characterized by a typical nodular structure exhibited by PA membranes formed by the reaction of PIP and TMC [62]. This dense layer formed by the cross-linking reaction of the monomers will contribute to good nanofiltration performance, which will be discussed in the subsequent sections. It is also worth noting that the control membrane M-0 exhibited a larger nodular structure, while membranes made from nanocomposites were characterized by relatively smaller nodular structures. This could be attributed to the improved hydrophilicity of the nanozeolite embedded supports that enabled uniform coating of the PIP solution. In addition, PA layer thickness gradually reduced from 280 nm to 191 nm due to improved cross-linking. Considering the fact that all the parameters for IP have been fixed, changes in the support properties would have contributed to the variation of the selective layer thickness and morphology. For example, it has been reported that increased wettability of the supports will positively contribute to a thinner selective later due to a higher cross-linking degree. These observations are in line with previous studies [27,34]. Further, to confirm the formation of the PA layer, the FTIR spectra of all the membranes were recorded to investigate the surface chemistry. Figure 6a shows a comparison of the spectrum obtained for all the membranes. All characteristic peaks corresponding to PSf were registered in all the spectrums, which are already discussed in Section 3.2. In addition to this, a sharp peak at 1624 cm^−1^ attributed to the amide bond is evidence of the successful formation of the PA layer [34].

Permeance and separation efficiency of all the membranes are presented in Figure 6. The membranes displayed a pure water permeance of 9.55 ± 0.6 Lm^−2^ h^−1^ bar^−1^, 11.15 ± 0.9 Lm^−2^ h^−1^ bar^−1^, 17.1 ± 2.1 Lm^−2^ h^−1^ bar^−1^, 13.8 ± 1.4 Lm^−2^ h^−1^ bar^−1^, 12.4 ± 1.6 Lm^−2^ h^−1^ bar^−1^ respectively for M-0, M-0.05, M-0.1, M-0.15, and M-0.2. Even the control sample offered a relatively high pure water permeance of 9.55 ± 0.6 Lm^−2^ h^−1^ bar^−1^ owing to the thin selective layer and probably due to the absence of non-woven support, which may have otherwise added extra transport resistance. Further, the permeance was observed to be following a trend similar to the support layers, where the highest permeance was observed for M-0.1. The permeability based on the known PA layer thickness showed an increase up to M-0.1 followed by a significant reduction thereafter, irrespective of the thinner selective layer (Supplementary Figure S9). This trend, similar to the permeance, highlights the influence of support layer resistance on the overall flux of the membrane. Accordingly, the increased permeance could be attributed not only to the thinner selective layer but also to the low resistance offered by the support layers with improved porosity. The efficiency of all the fabricated membranes for NF has been evaluated by conducting filtration experiments using aqueous salt solutions of NaCl, MgCl_2_, MgSO_4,_ and Na_2_SO_4_ with a concentration of 1000 ppm. Similar to typical NF membranes, all membranes offered significantly higher rejection to divalent salts relative to monovalent salts with a general trend in the order of Na_2_SO_4_ > MgSO_4_ > MgCl_2_ > NaCl. The highest rejection observed for NaCl was 28.4%, whereas MgCl_2_ was 57.4% for the case of M-0.2. Meanwhile, all membranes offered rejection above 90% for MgSO_4_ and Na_2_SO_4_. Selectivity of the membranes progressively increased with nanozeolite content in the support. Figure 6d shows the permeance of salt solution for different membranes where a considerable reduction in the flux was observed compared to pure water, which is consistent with the literature [63]. This behavior is typical of dense NF membranes and more pronounced for the divalent salt solution [64]. The addition of nanozeolites in the support is accompanied by an increase in the surface pore size, as explained previously. This would generally increase the possibility of defects in the PA selective layer. Nevertheless, we did not observe any such decline in the selectivity caused by the large pores on the support. This is possibly due to the fact that our substrate pore size is still smaller than 250 nm at the highest nanozeolite loading, which is considerably smaller than those that reported a reduction in the rejection of PA-based NF membranes due to large pores on the support [27].

### 3.4. Role Nanozeolites in Membrane Performance

The overall permeance of TFC membranes is directly influenced by several parameters that can be broadly classified into: (1) resistance from the support and (2) characteristics of the selective layer. Firstly, lower resistance from the support layer is favorable for improved permeance. Incorporating nanozeolites in the support layer improved the overall porosity, surface pore size, and hydrophilicity, thus enabling substrates with high permeability. Highly permeable support layers offer low transport resistance contributing to improved permeance. However, this increase in favorable properties of the support layer was only significant for up to a concentration of 0.1%, beyond which added nanozeolites lead to pore blockage. Secondly, changes in the surface properties of the supports by the incorporation of nanozeolites would have influenced the formation of PA layers to an extent. Improved hydrophilicity of the support layers by the incorporation of nanozeolites enabled the formation of a thinner selective layer which subsequently favored higher permeance of the composite membranes. Having said that, it is evident that the selective layer thickness was reduced by less than 30% for the most hydrophilic support with respect to the control sample. Meanwhile, a recent study has reported a significantly higher reduction in the selective layer by enhancing the wettability of the support layer [34]. Therefore, considering the fact that the membrane permeance displayed a trend similar to that of the support layers irrespective of the selective layer thickness, it is reasonable to conclude that the resistance from the support layer had a greater influence on the overall membrane permeance. Thus, supports with lower resistance displayed enhanced water permeance.

The selectivity of typical NF membranes is determined by electrostatic repulsion and steric hindrance [65,66]. From the obtained high rejection values of divalent salts, the role of steric hindrance is clearly evident. Divalent salts are characterized by a larger hydrated radius compared to monovalent ions, thereby adding the possibility of higher steric hindrance. For example, Mg^2+^ is shown to have a larger hydrated radius, while both Na^+^ and Cl^−^ ions with a smaller hydrated radius could easily pass through the membrane resulting in a poor rejection for NaCl [67]. It can also be seen that the increase in NaCl rejection is negligible for M-0, M-0.05, and M-0.1 despite considerable variations in the permeance and support properties. However, we could observe a noticeable increase in the rejection of NaCl for M-0.15 and M-0.2, which could possibly be due to the effect of nanozeolites. Firstly, the addition of nanozeolites can contribute to increased surface charge [39]. To confirm this, we determined the surface zeta potential of all the membranes at neutral pH, which is provided in Figure S10. As expected, the zeta potential of the membrane gradually increased, indicating a higher surface charge for M-0.15 and M-0.2, which could be attributed to the presence of higher nanozeolites, particularly on the surface of the support layer. On the other hand, the presence of nanozeolite beneath the PA layer might also contribute to additional rejection by size exclusion [68]. Although Y-type zeolites are characterized by pores in a range of 0.74 nm, which is significantly higher than the size of monovalent salts, studies have reported that faujasite structure can effectively filter hydrated Na^+^ ions. However, the role of nanozeolites beneath the PA layer in size sieving and electrostatic repulsion needs further investigation to determine the contribution of individual effects. In general, we can infer that rejection of our membranes is a combination of steric hindrance and electrostatic repulsion, where electrostatic repulsion is more expressed at higher nanozeolite concentrations.

## 4. Conclusions

In conclusion, we have successfully fabricated PA membranes on PSf supports modified with varying concentrations of nanozeolites. We demonstrated the possibility of tuning features of the membrane support layer by the addition of nanozeolites, thereby influencing the overall membrane performance. The incorporation of nanozeolites in the casting solution was found to influence the porosity, wettability, and pore size of the support layers, which subsequently influenced the performance of the NF membranes. All membranes maintained a high rejection towards divalent salts in general and a pure water flux of 17.1 ± 2.1 Lm^−2^ h^−1^ bar^−1^ at optimum substrate preparation conditions. While the presented results may not be conclusive enough regarding the role of unique pore structure and the smaller dimension of nanozeolites, future work should focus on comparing the effect of zeolite size and utilization of zeolite pores for enhancing the separation performance.

## Figures and Tables

**Table 1:** Composition of casting solution for all the supports expressed in mass percentage.

Membrane	PSf (%)	PVP (%)	Nanozeolite (%)	NMP (%)
S-0	17	3	0	80
S-0.05	17	3	0.05	79.95
S-0.1	17	3	0.1	79.9
S-0.15	17	3	0.15	79.85
S-0.2	17	3	0.2	79.8

## Data Availability

Not applicable.

## Supplementary Materials

The following supporting information can be downloaded at: https://www.mdpi.com/article/10.3390/membranes12100940/s1, Figure S1: Custom-made plate and frame set-up for membrane fabrication through interfacial polymerization; Figure S2: Schematic illustration of dead-end filtration system used for characterizing permeance of membrane supports and nanofiltration performance of PA membranes. Figure S3: Particle size distribution of nanozeolites obtained after ball milling. Figure S4: Left: Nitrogen adsorption isotherm and pores size distribution of nanozeolites. Right: Comparison of XRD patterns of nanozeolite and parent zeolite. Figure S5: EDS spectrum of all the supports fabricated with varying nanozeolite concentrations. Figure S6: Elemental mapping of Si atom distribution on the surface of membrane supports prepared with varying nanozeolite concentration (**a**,**b**) S-0, (**c**,**d**) S-0.05, (**e**,**f**) S-0.1, (**g**,**h**) S-0.15, and (**i**,**j**) S-0.2. Figure S7: Pores size distribution of membrane supports as observed from SEM images. Figure S8: FTIR spectra of all membrane supports. Figure S9: Permeabilities of different membranes. Figure S10: Zeta potential of PA membranes at neutral pH.
