# Supplementary material for: Fabrication of Thin Film Composite Membranes on Nanozeolite Modified Support Layer for Tailored Nanofiltration Performance
Source: Membranes (Basel). 2022 Sep 27;12(10):940. doi: 10.3390/membranes12100940 (PMC9610575; doi:10.3390/membranes12100940)
Supplement: Supplementary file 1 [file membranes-12-00940-s001.zip › membranes-1930087-supplementary.pdf]

Fabrication of thin film composite membranes on nano-zeolite modified support layer for tailored nanofiltration performance.

Shabin Mohammed[1], Haya Nassrullah[1,2], Jamaliah Aburabie[1], Raed Hashaikeh[1,] *

[1] *NYUAD Water Research Center, Department of Engineering, New York University Abu Dhabi, Abu Dhabi P.O. Box 129188, United Arab Emirates*

[2] *Chemical and Biomolecular Engineering Division, New York University, Tandon School of Engineering, NY, 11201, USA*

Corresponding author email address: raed.hashaikeh@nyu.edu

SUPPLEMENTARY DATA

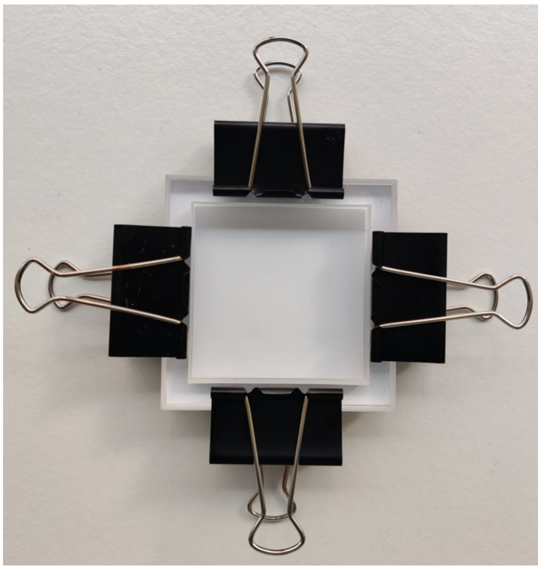

***Figure S1**: Custom-made plate and frame set-up for membrane fabrication through interfacial polymerization.*

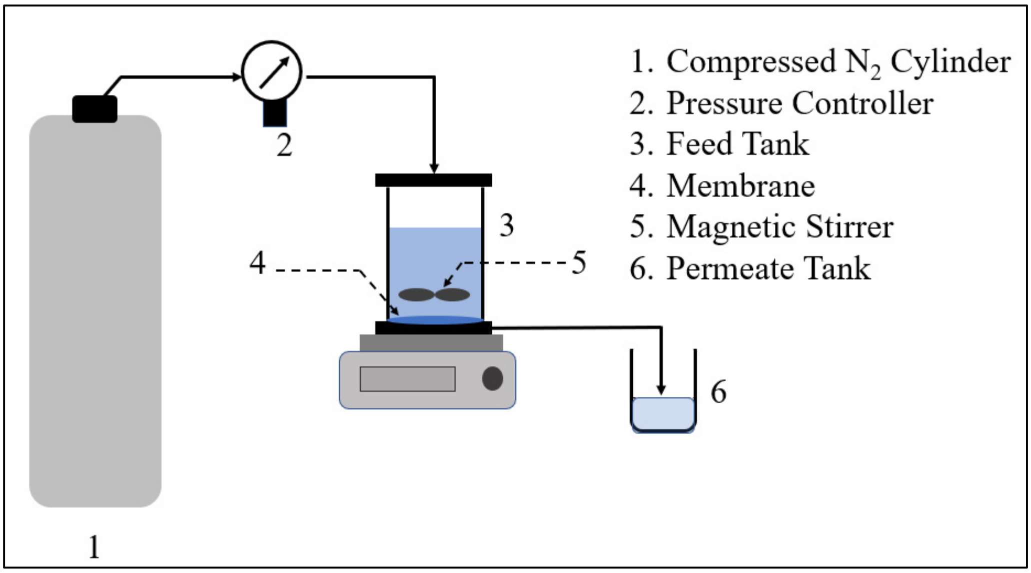

Figure S2: *Schematic illustration of dead-end filtration system used for characterizing permeance of membrane supports and nanofiltration performance of PA membranes.*

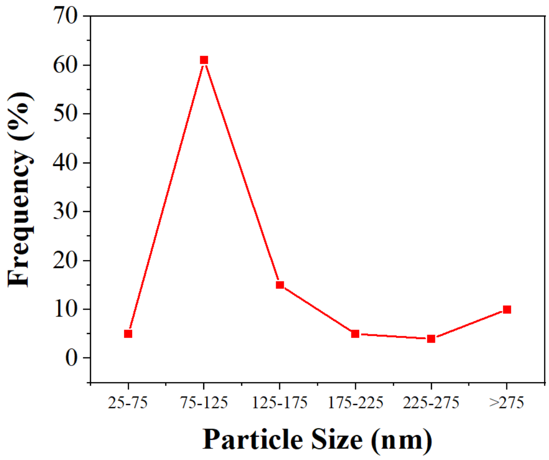

Figure S3: *Particle size distribution of nanozeolites obtained after ball milling.*

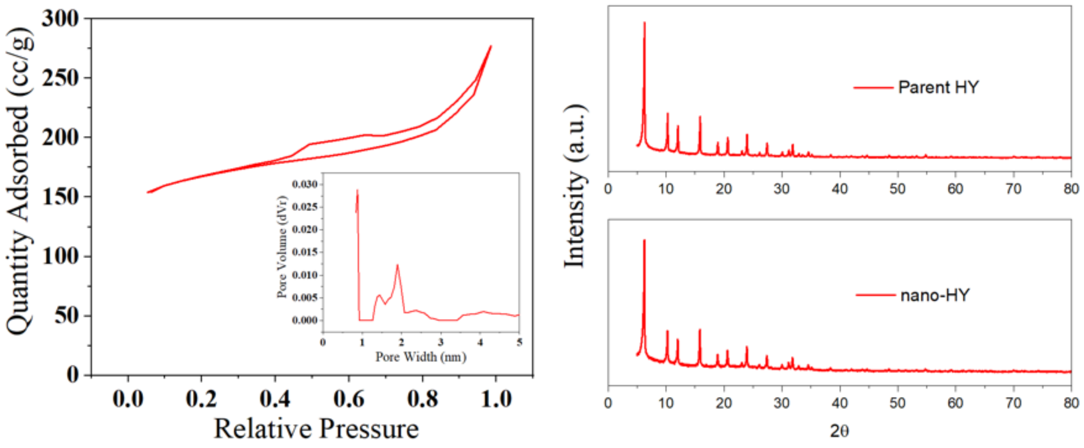

Figure S4: *Left: Nitrogen adsorption isotherm and pores size distribution of nanozeolites. Right: Comparison of XRD patterns of nanozeolite and parent zeolite.*

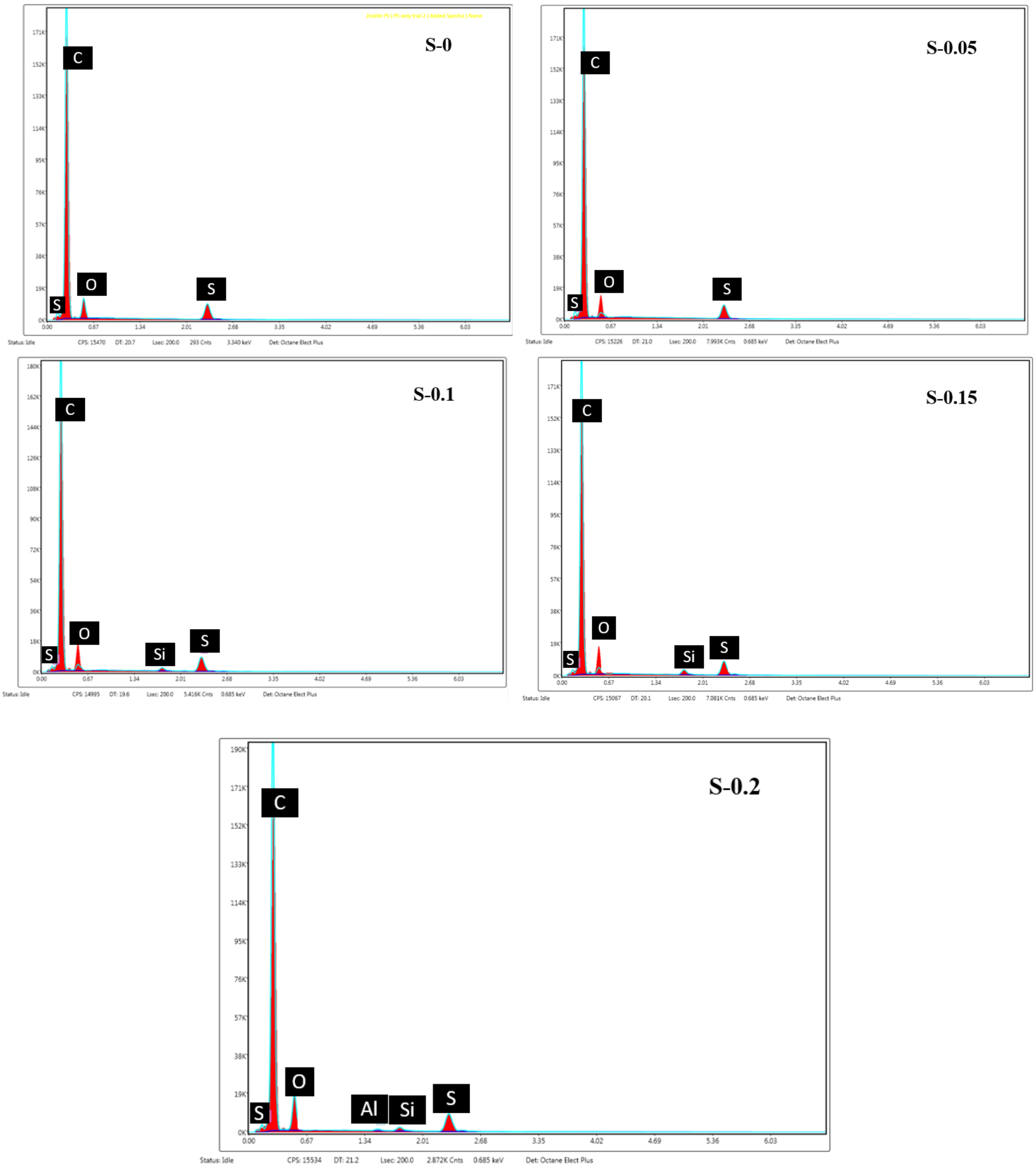

Figure S5*: EDS spectrum of all the supports fabricated with varying nanozeolite concentrations.*

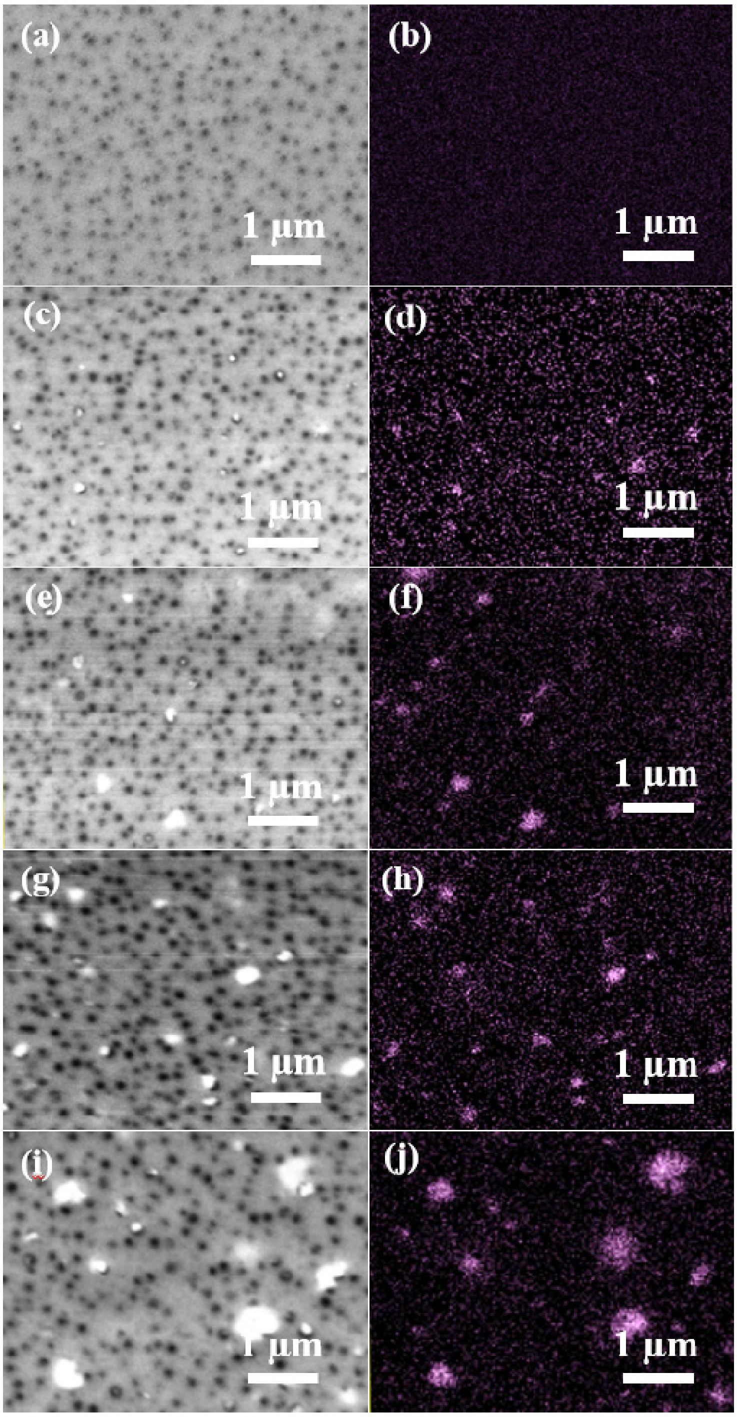

Figure S6: *Elemental mapping of Si atom distribution on the surface of membrane supports prepared with varying nanozeolite concentration (a,b) S-0, (c,d) S-0.05, (e,f) S-0.1 , (g,h) S-0.15 and (I,j) S-0.2*

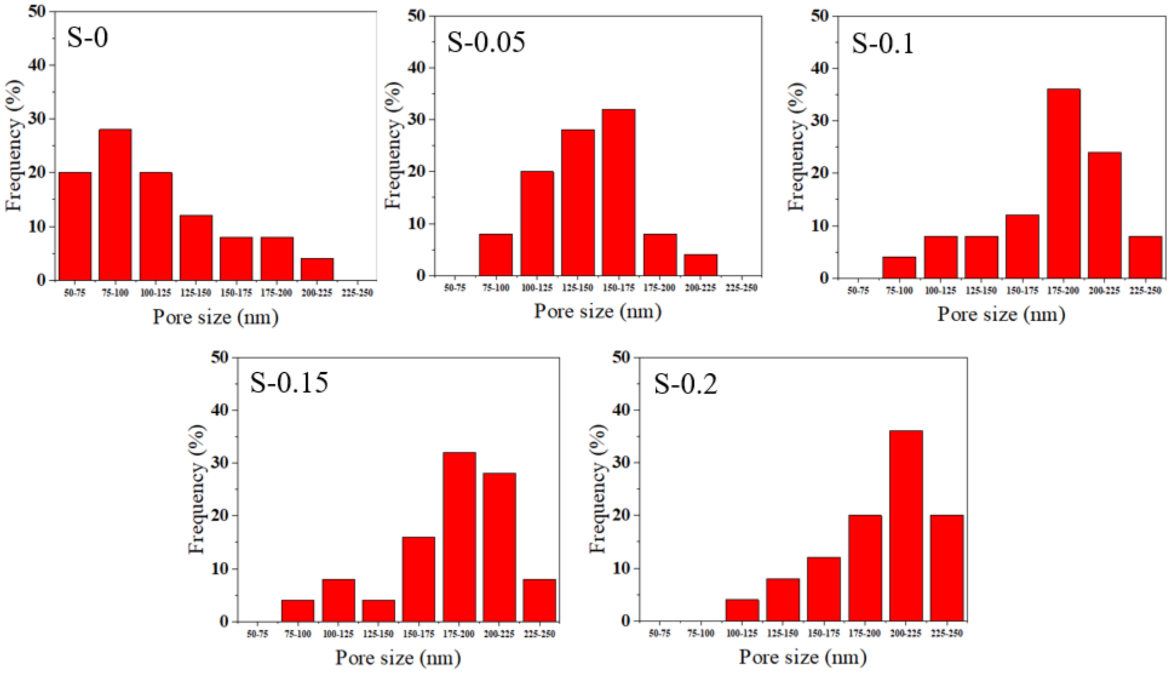

***Figure S7**: Pores size distribution of membrane supports as observed from SEM images.*

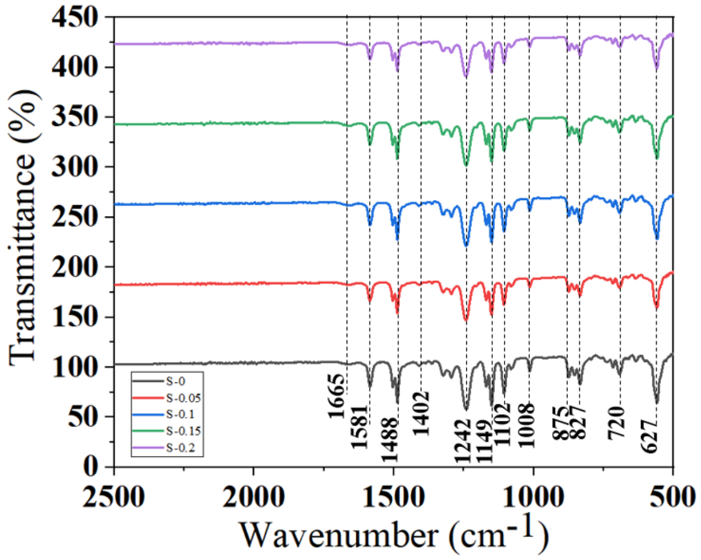

***Figure S8**: FTIR spectra of all membrane supports.*

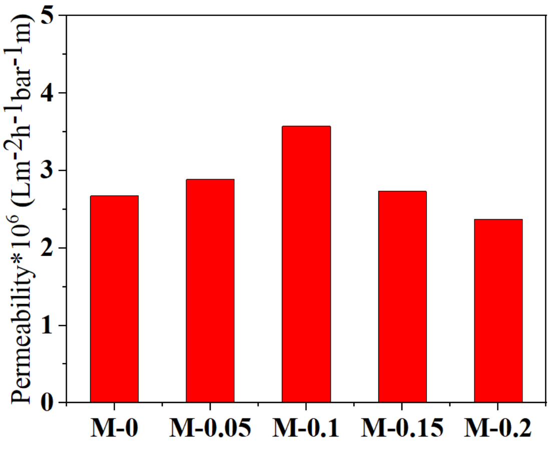

Figure S9: *Permeabilities of different PA membranes.*

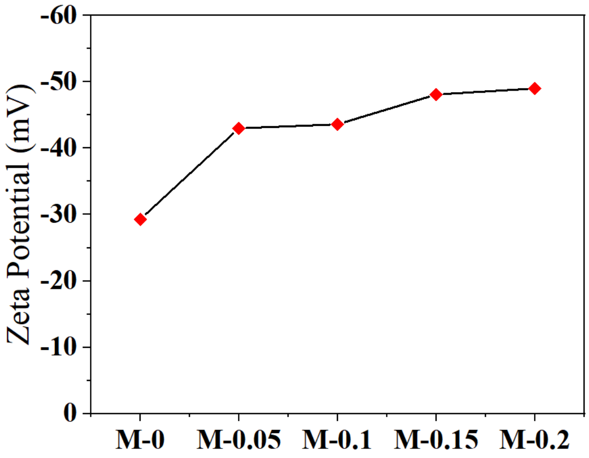

Figure S10: *Zeta potential of PA membranes at neutral pH.*
